# Defining High-Risk Patients Suitable for Incisional Hernia Prevention

**DOI:** 10.3389/jaws.2023.10899

**Published:** 2023-02-03

**Authors:** Jose Antonio Pereira-Rodríguez, Alejandro Bravo-Salva, Núria Argudo-Aguirre, Sara Amador-Gil, Miguel Pera-Román

**Affiliations:** ^1^ General and Digestive Surgery Department, Parc de Salut Mar, Hospital del Mar, Barcelona, Spain; ^2^ Department of Medicine and Life Sciences, Pompeu Fabra University, Barcelona, Spain; ^3^ General and Digestive Surgery Department, Hospital de Granollers, Granollers, Spain

**Keywords:** prophylactic mesh, incisional hernia prevention, risk factors, small bites, laparotomy complications

## Introduction

There is a 9%–20% incisional hernia (IH) rate 1 year after midline laparotomy (1, 2) increasing up to 22.4% after 3 years of follow-up (3). Several prospective studies (4–7), metanalyses (8, 9) and guidelines (10) advise or have demonstrated that the use of prophylactic mesh (PM) reduces IH. Despite all these studies, the use of PM has not been spread worldwide (11). Among other reasons, this is because it is unknown for which patients the potential benefits outweigh the risks of complications when using a PM. Likewise, there are several concerns among surgeons regarding which complications can occur using a PM (remarkably chronic pain and infection) (12). Due to these, it is necessary to determine diseases, patients and situations where high risks of IH justify consideration of using a PM.

This paper aims to review as an opinion article the scientific data on situations, patients and diseases with a higher risk of IH in which PM should be considered.

## High-Risk Related Situations

### Emergency Laparotomy

In almost all studies focused on risk factors for IH, emergency laparotomy has a higher risk of IH than elective laparotomy. In two studies comparing emergency to elective laparotomy, a Hazard ratio (HR) of 2.31 (13) and a Odds ratio (OR) of 4.71 (14) respectively were demonstrated. This risk can be even worse in patients when other risk factors are present at the emergency laparotomy (15). In presence of peritonitis IH can reach 50% (16) or when an ostomy is associated the risk is 6 times increased (OR 5.8; *p* = 001) (17).

Systematic abdominal wall closure with small bites (SB) technique significantly reduces fascial dehiscence (FD) (6.6% vs. 3.8%) 6 and IH (27% vs. 15%) (18) in emergency laparotomy. Moreover, the use of PM in these situations, especially in the presence of other risk factors, reduces even more the incidence of FD and IH (19–21).

### Redo Laparotomy/Early Abdominal Reoperation

Reoperation during the same episode due to surgical complications is one of the worst situations in terms of development of IH. Some studies have shown incidence rates even higher than 50% after both emergency (20) and elective (22) surgery, alsodemonstrating that in this scenario using a PM can reduce IH incidence (20).

In the external validation of the HERNIA score (23), patients with earlier abdominal operation had an IH incidence of 55.3%, and this factor was added in the formula with 3 points (high risk group patients were defined as > 9 points).

A previous laparotomy is also an IH risk factor, though it is not comparable to reoperations in terms of IH incidence and does not influence FD.

### Ostomy

The creation of an ostomy during midline laparotomy has been pointed out as a high-risk factor for IH development (24). Timmermans et al. found ipsilateral rectus abdominal muscle atrophy to the ostomy as the main cause of IH formation in those patients. Moreover, the study underlined high rates of IH: 37% if diagnoses were by physical examination and 48.3% with CT scan. This also highlights that the incidence of IH can reach up to 58% when it was performed as an emergency midline laparotomy (OR 5.8%; *p* = 0–016) (17).

### Contamination Grade

The contamination grade and its correlation to IH is another risk factor associated, probably due to the high risk of development of a wound infection (13, 25).

In an observational study with a large cohort of patients, CDC wound grades III or IV (13) were associated with an increased risk of IH in univariate OR 2.29 (*p* = 0.001) and multivariate analysis HR 2.26 (*p* = 0.001).

In fact, all the risk situations described above (emergency, redo laparotomy and ostomy) are commonly associated with higher grades of contamination (26).

## High-Risk Factors Related to Patients

### Age

Elderly age emerges as risk factor for IH and FD in several studies both in univariant and multivariant analysis (HR 1.30 for every 10-years increase) and HR of 2.96 in patients older than 70 years for FD (13). When age has been analysed as an isolated risk factor after midline laparotomy only, there were statistically significant differences in long-term outcomes when age was over 75 years old (27).

In our opinion, the patient’s age as an isolated data to decide on using a PM is not enough. We should consider other associated risk factors and elderly age would probably act as an indirect indicator of patients’ health status.

### Obesity

Obesity is a well-known risk factor and correlates directly with IH. There is a large number of studies describing the role of BMI over 25 kg/m^2^ as an IH risk factor and this appears as one of the items used to evaluate for the majority of predictive scores (14, 22, 23, 28). BMI is a deciding factor regardless of other factors when considering using a PM, given its high association with IH incidence (mainly over 30 kg/m^2^) when performing a midline laparotomy. In one study (13), univariate analysis was associated with 2.29 OR (IC 95% 1.5–3.51; *p*= < 0.001) when the patient was overweight (BMI 25–30 kg/m^2^) and 2.81 (IC 95% 1.42–5.52; *p* = 0.002) when BMI > 30 kg/m^2^. Multivariate analysis showed an increased HR of 1.76 (*p* = 0.001).

Studies investigating prophylaxis (29) showed a decrease in IH incidence after midline laparotomy when the patient had > 30 kg/m^2^ BMI (76%–13%; *p* = 0.001).

### Smoking

Tobacco consumption due to its wound healing alterations and direct relation to chronic obstructive pulmonary disease (COPD) is one of the risk factors detected in many IH risk investigations (26). However, other studies have shown no relation as an independent risk factor for IH (13). Again, in our opinion, smoking without other associated risk factors cannot be considered alone to decide on using prophylactic measures after midline laparotomy.

### Nutritional Status

It seems that malnutrition should be a prognostic factor for IH. However, there is a lack of studies comparing categorically nutritional status, albumin blood levels and IH risk. Therefore, there is no solid evidence to consider the nutrition status as a parameter to predict IH development.

### Collagen Diseases (Abdominal Aortic Aneurism)

The high rate of association of IH after midline laparotomy in collagen disease patients related to abdominal aortic aneurism (AAA) has been widely demonstrated in studies of high scientific evidence (30–32). In this scenario, IH can reach up to 30%–60%. However, in a large study on risk factors (13) it did not show statistical significance.

In the studies on using PM after open AAA repair, a significant reduction (49.2% vs. 0.0%) was demonstrated when a PM was used in a retromuscular plane (33, 34).

Therefore, the presence of an AAA in every open procedure should be considered alone as an indication on using a PM even if there are no other risk factors associated.

### Associated Morbidity

A high number of comorbid conditions, such as hypertension, diabetes mellitus, COPD, heart disease, cancer, depression and hepatopathy have been related to IH (14, 35).

As a single risk factor, no one of them seems to have enough power to decide on using prophylactic measures. In the multivariate analysis of the Itatsu et al. study, no relation of any associated comorbidity showed a statistically significant relation with IH. Nevertheless, in the development of a predictive IH model (14), more than two Elixhauser comorbidities, COPD, ASA status, cancer and liver disease were associated with a higher risk of suffering an IH.

## High-Risk Pathology

### Resection of Intra-abdominal Malignancy

Cancer surgery has a significantly higher risk of IH (OR 1.25; *p* = 0.003) (14). Moreover, previous oncological surgery (13) (HR1.33; *p* < 0.001) and metastatic cancer (OR 0.77 *p* = 0.0009) (35) have both been revealed as risk factors in univariant analysis.

In a major study investigating IH incidence in patients surviving after surgery for abdominal malignancy, where 1,847 CT scans from 491 were revised (36), 41% of occurrences of IH were diagnosed with an incidence range between 23% (after nephrectomy) and 62% (after hepatectomy).

### Colorectal Surgery

Colorectal surgery is one of the most common risk factors for IH found in most studies. After colorectal surgery the incidence of IH can reach between 35% and 50% (35–37) with also undesirable rates of FD (3.9%–5.2%) (38).

In the research to create a score for FD (25), colorectal surgery showed the highest incidence (5.2%) and in the final score system receives 1.4 points of a total of 10.6.

In the univariate study, compared to other gastrointestinal operations, colorectal surgery is the one with the highest association to IH risk (OR 1.83; *p* < 0.001) (13) though without reaching statistical significance in the multivariant analysis.

The relationship with higher IH incidence would be probably a consequence of other comorbidities or situations that are present in patients (elderly age, wound contamination, and surgical site infection) acting as IH risk factors. Colorectal surgery is the most common type of surgery related to wound complications both in univariant (OR 7.08) and multivariant (OR 3.21) (26).

Some researchers (29, 35) have focused on using predictive scores or algorithms to select suitable patients for PM use, showing good results in terms of IH (OR 7.58; *p* > 0.0001) (35) and FD (4.6% vs. 0%; *p* = 0.03) prevention (29). Comparable results have been demonstrated in a randomized control trial of both elective and emergency colorectal resection, where the IH relative risk reduction of 62% and an absolute risk reduction of 22% when using PM after midline laparotomy.

### Liver Transplantation

Accumulated incidence after liver surgery can reach up to 27% after 72 months of postoperative follow-up (39). When looking specifically at liver transplantation, remarkably, IH is one of the most common long-term complications with an incidence of between 5% and 40% (40, 41). Due to the comorbidities in patients with terminal liver diseas, these patients have several risk factors for IH development (42). Also, the treatment with immunosuppressors increases the risk of IH and surgical site complications (43, 44). All these facts provide patients with an important decrease in quality of life (41).

### Bariatric Surgery

Incidence of IH after bariatric surgery has been reported to be as high as 25% (45) and 50% in superobese patients (46). PM has proven to be effective and safe in two randomized control trials (47, 48) and one metanalysis with a global reduction to a third of the risk for IH (OR 0.30; *p* = 0.004) (49).

## Scores Systems

Due to the heterogeneity of the risk factors and the difficulties involved in standardizing the decision making, some authors have designed predictive models using score systems to evaluate the tailored risk of IH and FD. The main concern with some of these scores is the use of postoperative variables in the calculation, which reduces the potential of the scores to help the surgeon in the pre or perioperatively decision process and only helps to advise the patient, implement prehabilitation or maintain longer follow-up in risky patients.

### HERNIAscore

The Hernia score (28) was created using a cohort of 625 patients with a median follow-up of 42 months. Independent predictive factors detected in this study were: laparotomy or assisted laparoscopy, COPD, and BMI. By using the equation: 4*laparotomy + 3*HAL+1*COPD+1*BMI ≥ 25, three risk groups were created: low risk (0–3 points), 5.2%; moderate (4–5 points), 19.6%; and several risks (more than 6 points), 55%.

Afterward, the Hernia score was modified and validated using a new equation where a previous laparotomy was added to it: 1*(BMI≥25) + 1*(COPD) + 5*(extended laparoscopy) + 6*(laparotomy) + 3*(earlier abdominal operation). Risk groups were defined as: low risk (score 0–6.9 points) 6.9%; medium risk (7.0–9 points), 35.6%; and high risk (≥9 points), 57.5% IH incidence.

### PENN Hernia Risk Calculator

By using a database of 78,030 patients from 3 high-volume hospitals in Pennsylvania, 558 variables were analyzed in 29,739 eligible patients. Data from a group that needed IH repair with those who did not were compared (14). As a result, an individualized model using 16 variables (type of surgery, age, race, BMI, surgical and pathological characteristics) was designed. Related to the risk, four groups were created: low, medium, intermediate, and high risk.

### Other Scores

One of the first attempts to develop a predictive score was focused on predicting abdominal wound dehiscence (25). This score used preoperative and postoperative characteristics, hindering the application from preventing, or from helping with decision-making regarding, PM use. This risk model using only preoperative characteristics was applied to a 176 patients’ cohort without reaching predictive values (50).

In a retrospective study on colorectal surgery where 30,741 patients were included, an actionable model of IH prediction was produced. The groups generated were: low (3.9%); moderate (7%); high (12.6%); and extreme risk (19.8%). It is interesting to point out that 30% of patients included in the study were from high and extreme high-risk groups which indirectly shows the high probability of IH after colorectal surgery.

In a prospective study with 332 patients analyzed after open surgery for colorectal cancer (31), an algorithm including patients’ BMI and risk factors for IH was analyzed to help surgeons with decisions on PM use. As a result, the proper use of the algorithm decreased the incidence of IH (OR 4.41; *p* < 0.001).

## Discussion

The development of an IH is a major problem after abdominal surgery. It correlates with a decrease in patients’ quality of life, frequently needs repairing, and produces an increase in healthcare costs (51, 52).

To decrease the incidence of IH with prevention seems a key issue. Thus, to provide tools enabling the surgeons, before the operation, to individualize and advise the risk of IH to the patients may help surgeons and patients make a shared decision regarding the best prevention strategy.

From our point of view it is remarkable that there are situations that by themselves need special attention: emergency surgery, redo laparotomy, contaminated surgery, and ostomy creation.

Emergency surgery has a high risk of IH that is even higher when other risk factors are combined. The analyzed studies, despite their low quality of evidence, demonstrated that a PM prevents both FD (19) and IH (20). A well designed prospective and randomized study seems essential.

Redo laparotomy has been poorly investigated and clearly demands high quality studies to confirm it as a high-risk group and to define the best prevention strategy.

Contaminated surgery, due to the high frequency of wound infection in CDC grades III and IV (12%–20%) (53) and the association with IH development, is the most controversial situation. Although we have some evidence regarding the safety on using a mesh in contaminated fields (20, 29, 54, 55), many surgeons are reluctant to use a PM for the risk of prostheses infection (12).

In our opinion, when closing a laparotomy during a surgery that is an emergency, redo, contaminated, or associated with an ostomy, two data points should be considered: the contamination grade and the patient’s risk factors. In a contaminated or infected operation with a controlled sepsis focus in a patient with associated risk factors for IH, we recommend considering using a PM to prevent FD and IH as well. At least, if PM is not used, surgeons should try to accurately close the laparotomy. Nevertheless, the scientific community needs to pay attention and provide higher evidence quality studies on this important issue.

Regarding patient risk factors: obesity and AAA have enough evidence to strongly suggest, if the situation allows it, the use of a PM (33, 34) to prevent IH even in the absence of associated risk factors.

Individually, the rest of the risk factors analyzed do not have such a strong association with IH to recommend PM use when present. Nevertheless, some authors have demonstrated that the presence of several risk factors at the same time increases the predisposition to develop IHs. This presumes a summative effect of risk factors and, from our point of view, when two or more risk factors are present, using a PM may be justified.

In cancer, colorectal, transplantation or bariatric surgery, special concern must be taken when performing the laparotomy. A tailored approach should be utilised with these patients considering their IH risk factors and considering the use of one of the predictive scores mentioned above can be useful. Thus, we believe that in elective surgery a careful analysis should be taken to choose IH preventive measures like avoiding midline incisions, performing SB technique, or using a PM, as it is also suggested in the EHS guidelines on abdominal wall closure (10).

The SB closure technique should be the selected technique for all midline elective laparotomies, given current evidence in the literature. Some studies have demonstrated the effectiveness and safety of SB use in reducing IH (4, 5). However, there is a recent randomized prospective study (56) where no statistical significative difference in IH reduction after 1-year follow-up was reached (3.3% vs. 6.4%; *p* = 0.173). Notwithstanding, when FD was added to IH, the difference was considered statistically significant (4.8% vs. 11.3%; *p* = 0.018). In another study, performed in low-risk IH patients (57) with 2-years follow-up, lower IH incidence in the SB group was revealed without statistical differences (3.6% vs. 12.1%; *p* = 0.20). The same authors performed another study in high-risk patients (58), demonstrating that when using PM after a median follow-up time of 29.3 months, IH incidence decreased (HR 11.79; *p* < 0.0001) independently of the closure technique (small or large bites). They also outline that the worst results were obtained when laparotomies were closed with neither SB nor PM.

It is notable that predictive scores developed up to now (14, 23, 25, 28, 35) have some limitations, for example they have been studied in retrospective cohorts, and one study (14) calculated IH as only those patients who needed a repair, as a result the real incidence was probably underestimated. Moreover, all of them have been created to predict IH and not to help on the decision to use a PM. With all this information, in our opinion, predictive scores only can be used as a guidance tool to help in patients’ shared decision process or with research.

In conclusion, there are different situations, types of operation or patients who have a higher risk of developing an IH. Emergency, redo, contaminated or ostomy association, midline laparotomies; obesity, AAA, two or more comorbidities; cancer, colorectal, transplantation and bariatric surgery, have a high risk of IH. Predictive score and considering surgical characteristics provide us with a guide to select the best approach, the best closure technique or whether or not to use a PM, and can help to share the decision making process with our patients.
